# Individual factors predict substance use treatment course patterns among patients in community-based substance use disorder treatment

**DOI:** 10.1371/journal.pone.0280407

**Published:** 2023-01-12

**Authors:** Evangelia Argyriou, Giorgos Bakoyannis, Wei Wu, Mary Jo Rattermann, Melissa A. Cyders

**Affiliations:** 1 Department of Psychology, Indiana University—Purdue University Indianapolis, Indianapolis, Indiana, United States of America; 2 Department of Biostatistics and Health Data Science, Indiana University Fairbanks School of Public Health and School of Medicine, Indianapolis, Indiana, United States of America; 3 Fairbanks Addiction Treatment Center, Indianapolis, Indiana, United States of America; The Ohio State University, UNITED STATES

## Abstract

**Background and objectives:**

Substance use disorders (SUDs) usually involve a complex natural trajectory of recovery alternating with symptom reoccurrence. This study examined treatment course patterns over time in a community SUD clinic. We examined depressive symptoms level, primary SUD assigned at each admission, and lifetime misuse of multiple substances as potential risk factors for premature treatment termination and subsequent treatment readmission.

**Methods:**

De-identified longitudinal data were extracted from charts of 542 patients from an SUD treatment center. Survival analysis methods were applied to predict two time-to-event outcomes: premature treatment termination and treatment readmission.

**Results:**

Primary opioid (vs alcohol) use disorder diagnosis at admission was associated with higher hazard of premature termination (HR = 1.91, p<0.001). The interaction between depressive symptoms level and substance use status (multiple vs single use) on treatment readmission was significant (p = 0.024), such that higher depressive symptoms level was predictive of readmission only among those with a history of single substance use (marginally significant effect). Lifetime use of multiple (vs single) substances (HR = 1.55, p = 0.002) and age (HR = 1.01, p = 0.019) predicted increased hazard of readmission.

**Conclusions:**

Findings did not support a universal role for depressive symptoms level in treatment course patterns. Primary SUD diagnosis, age, and history of substance misuse can be easily assessed and incorporated into treatment planning to support SUD patients and families. This study is the first to our knowledge that afforded a stringent test of these relationships and their interactions in a time-dependent, recurrent event, competing risks survival analysis examining both termination and readmission patterns utilizing a real-world clinic-based sample.

## Introduction

Substance use disorders (SUDs) involve a complex trajectory of recovery alternating with symptom reoccurrences [1, 2]. This is partly reflected in patterns of repeated treatment readmissions, which are associated with greater disruption to patients and their families and higher healthcare costs [3]. Premature treatment termination further contributes to these patterns by accelerating subsequent symptom relapse [4, 5]. It is essential to evaluate risk factors of premature SUD treatment discharge and readmission patterns to design more effective treatment and improve outcomes. A variety of risk factors for premature treatment termination and readmission have been identified in the literature (although not consistently) including systematic and social factors often driven by external variables, such as type of treatment modality [3], homelessness [6], health insurance [6], unemployment [7], as well as individual factors which could be more effectively used in treatment selection and planning, including demographics (primarily age), cognitive deficits, treatment alliance, and psychiatric comorbidity [8]. Depressive disorders are often comorbid with or secondary to SUD, predict a more severe and complicated SUD presentation [9], and may interfere with SUD treatment, reducing treatment completion and increasing the risk for relapse [10–12]. Higher depressive symptoms level could complicate clinical diagnosis and treatment [13] by impeding rapport and engagement in the treatment process, resulting in premature treatment attrition [14, 15] and accelerated treatment readmission through accelerating relapse [2, 10]. Depressive symptoms predict more subsequent treatment readmissions [11, 12] but may also decelerate readmission by impeding motivation to seek treatment [16]. The relationship between depressive symptoms level and SUD treatment outcomes has varied across studies [15, 17–23], suggesting the need to examine moderators of this relationship.

The effect of depressive symptoms level on treatment course may differ across primary SUD and lifetime substance misuse history. Evidence suggests that opioid use predicts increased dropout rates [24–26]; however, these findings have not been consistent and depend on the definition of substance use (e.g., diagnosis or general misuse) and samples studied [8]. Illicit substance use disorders are more strongly linked to depressive symptoms [27, 28] and are associated with distinct patterns of time to relapse and treatment retention [14, 29]. Misuse of multiple compared to single substances or presence of multiple SUDs is associated with increased likelihood of depression [27] and readmission to treatment after discharge [17].

Previous studies have primarily examined the number of readmissions in a specific time period, drop-out rates, or treatment length [11, 12]. This fails to appreciate the aspect of time on treatment course, the interaction between depressive symptoms level or substance use variables over time, and more complex aspects of treatment course (e.g., reasons for termination). Baseline measures of depressive symptoms level and substance use often represent the previous two weeks of symptoms and may not track very closely with subsequent changes more proximal to later treatment admissions. Treatment length should be considered in the context of discharge reason: Discharge after successfully completing treatment may be underpinned by different processes than discharge due to premature termination. Collapsing across successful and premature termination masks important differences and prevents examination of unique underlying mechanisms.

The current study examined discharge and readmission over time in a community-based SUD clinic. We evaluate three *individual* factors that are commonly assessed in community-based clinics and readily available for treatment planning without additional burden: depressive symptoms level at each admission, primary SUD at each admission, and lifetime misuse of multiple substances. The current study makes several unique contributions rarely seen in previous literature. First, we examined both primary SUD and lifetime misuse of multiple substances in the same model, controlling for the effect of each to the other, in order to determine which may be more important to consider in treatment planning. Second, we assessed the interactions between depressive symptoms level and substance use variables, controlling for demographic variables previously found to predict these SUD treatment outcomes [8]. Given the low variance of other SUD diagnoses outside AUD and OUD in our sample, we focused on these two for our hypotheses. Treatment for OUD is significantly different in nature than that for AUD and this may be one mechanism driving premature treatment termination among those with OUD and subsequent readmission [30]. Third, we examined SUD treatment outcomes using survival analysis, which enables assessing the instantaneous rate of treatment completion and readmission and adjusts for the varying follow-up lengths across patients. Fourth, we analyzed treatment discharge using advanced completing risks analysis, which jointly estimates the *probability* of discharge type of interest (i.e., premature termination) and *time* to discharge from any cause. Finally, we analyzed all observed admission and treatment events over time.

We hypothesized:

1. Higher depressive symptoms level at admission, OUD (vs AUD) at admission, and history of misuse of multiple substances (vs single substance misuse) would predict increased hazard of premature termination.
2. Higher depressive symptoms level at admission, OUD (vs AUD) at admission, and history of misuse of multiple substances (vs single substance misuse) would predict increased hazard of treatment readmission.
3. Depressive symptoms level at admission would interact with the two substance use factors to predict treatment course, such that for those with OUD (vs AUD) at admission and history of misuse of multiple substances (vs single substance misuse), the association between depression level and premature termination and readmission would be stronger.

## Method

### Participants

De-identified longitudinal data were extracted from the charts of patients admitted to an SUD treatment center at least once between October 2014 and April 2018. This treatment center is one of the state’s most comprehensive systems for treating substance use and mental health disorders offering inpatient (detox, rehab, and residential treatment), outpatient (including intensive outpatient, partial hospitalization programs), long-term residential programs, and recovery housing. The final sample included charts from 542 patients (51.3% male, 88.2% Caucasian) between the ages of 15 and 72 (Mean = 36.5, standard deviation [SD] = 13.9) at their first intake to the clinic. Given the small percentage of patients with non-AUD or OUD diagnoses, these were collapsed into one category for analysis (i.e., ‘Other’).

### Measures

#### Demographics

Upon intake to the clinic, data were collected about patients’ sex assigned at birth, age, reported substances misused during one’s lifetime, and SUD diagnosis. SUD variables were assessed via a structured clinical interview performed by licensed mental health professionals.

#### Treatment completion and readmission

Although included charts indicated at least one admission between October 2014 and April 2018, data extracted included *all* prior treatment admissions for each individual. Data included dates of intakes and discharges, program(s) of treatment attended, SUD diagnosis at *each* admission, and reasons for discharge for *each* treatment admission to the clinic. Reasons for discharge were collapsed into two categories: successful completion and premature termination (including leaving against medical advice, being expelled, and medical reasons). After data compilation, we calculated the length of each treatment admission and the length of time to the subsequent treatment admissions for each treatment admission and discharge. Length of treatment was defined as the time period, in number of days, that patients remained continuously enrolled in the treatment facility receiving a sequence of inpatient and/or outpatient treatment programs for a given primary diagnosis. Length of time to the subsequent treatment readmission was defined as the time period, in number of days, from previous discharge to subsequent treatment readmission.

#### Patient Health Questionnaire—9 (PHQ-9)

The PHQ-9 was used to measure depression level over the previous two weeks at each admission to the clinic before initiating treatment. The PHQ-9 is a self-report measure consisting of 9 items based on the DSM–IV diagnostic criteria for Major Depressive Disorder. Items are on a four-point Likert scale (0 = not at all, 1 = several days, 2 = more than half the days, 3 = every day). Depression level is calculated by summing up item scores (range 0–27), with higher scores indicating higher depression level. Scores of 0–4 indicate no depression symptoms, 5–9 mild depression, 10–14 moderate depression, 15–19 moderately severe depression, and 20–27 severe depression. Validity of the PHQ-9 has been established using both community [31] and clinical samples [32].

### Procedures

During one admission between October 2014 and April 2018, all patients were offered the opportunity to allow their data to be utilized in a separate, larger study. Patients were told that their refusal to participate would not influence their treatment at the center. Adults provided consent and patients under 18 years old at admission provided assent, with consent provided by a parent/guardian. At each intake admission, patients completed the PHQ-9 as part of regular clinical care, which was also recorded in their charts. The data for the current analyses were compiled from these charts and were de-identified before being shared with the research team; thus, the project was deemed exempt from institutional board review by the local university. No previous papers have been published from these data.

### Data analysis plan

All analyses were conducted using the R statistical software. To capture the treatment course as a function of time we applied survival and competing risks analyses using the *survival* package. These methods were used to predict the two time-to-event outcomes of interest: premature treatment termination and treatment readmission.

#### Competing risks analysis for premature treatment termination

To estimate the effect of the time-dependent depression level (i.e., depression reported at *each* admission intake), primary SUD diagnosis (OUD vs AUD vs Other) at *each* admission, and lifetime misuse of multiple substances (vs single substance misuse), as well as the interactions between these variables, we implemented a competing risks survival analysis. The outcome in this analysis was the *instantaneous rate* (i.e., hazard) *of premature termination*. This rate reflects both the *probability* of the discharge type of interest (i.e., premature termination) and the *time* to discharge. Thus, this analysis incorporates both the likelihood of the discharge reason *and* the time to discharge, of each treatment admission for each individual, into one analysis. We also incorporated repeated measurements of depression level and primary SUD diagnosis at *each* treatment admission as predictor variables, allowing for the analysis of richer within-individual observations. The goal of our competing risks analysis was to estimate predictor and interaction effects on the instantaneous rate of transitioning from the initial “in treatment” state to the “premature termination” state, which was the main event of interest. A hazard ratio (*HR*)>1 for “premature termination” indicates that increase on a continuous predictor (e.g., PHQ-9) or a certain group in a categorical variable over the reference group (e.g., multiple vs single misuse) is associated with an increased rate (or hazard) of premature termination.

First, we estimated the cumulative incidence of premature termination (i.e., cumulative probability of premature termination over time) using a moment-based nonparametric estimator that takes into account the potential association across different treatment episodes of the same individual [33]. We created cumulative incidence plots to display the probability of premature termination after admission across depression and substance use subgroups over time. Two-sample nonparametric comparisons of the cumulative incidences were conducted using a Kolmogorov-Smirnov-type test [34]. To take the within-individual dependence into account, significance level was calculated using 1000 cluster bootstrap replications [33]. For the pairwise comparison of the cumulative incidences across the SUD groups (primary AUD vs primary OUD vs primary Other), we applied the Bonferroni correction. The multivariable analysis was based on the semiparametric proportional cause-specific hazards model. To take the within-individual dependence into account, standard errors were estimated using a robust sandwich variance estimator. All analyses were weighted using the inverse of the number of episodes for a given individual (Bakoyannis, 2021) to adjust for potential informative cluster size (e.g., that individuals with more episodes might be more prone to a premature discharge due to treatment burden).We tested potential nonlinearity of the depression effect by including a quadratic term of depression in the model. This allows us to model U or inverse U type associations, which are common in psychological phenomena, but rarely assessed. In this analysis, we first performed a global Wald test for the null hypotheses that no covariates are associated with premature treatment termination, which is not prone to Type I error inflation due to multiple comparisons. Hypothesis tests for individual covariate effects were interpreted only if this global test was statistically significant.

#### Survival analysis for treatment readmission

To estimate the effect of the time-dependent depression level, primary SUD diagnosis at each admission, and lifetime misuse of multiple substances (vs single substance misuse), as well as their interactions, we conducted a survival analysis using the semiparametric Cox proportional hazards model. The goal is to estimate the rate of transitioning from the initial “treatment discharge” state to “treatment readmission” state. The outcome in this analysis was the instantaneous rate of treatment readmission (i.e., hazard of treatment readmission). A *HR*>1 indicates that increase on a continuous predictor (e.g., PHQ-9) or a certain group in a categorical variable over the reference group (e.g., AUD vs OUD) is associated with increased rate of treatment readmission.

First, we estimated the cumulative incidence of readmission using a moment-based nonparametric estimator that takes into account the potential association across different treatment episodes of the same individual [33]. We created cumulative probability plots to display the probability of readmission after a discharge across depression and substance use subgroups over time. To account for repeated readmissions within each patient, we used the gap time formulation of the conditional risk set Cox model. We evaluated potential nonlinearity of the depression effect by including a quadratic term of depression in the model. Similar to the analysis of premature treatment termination, we first performed a global Wald test for the null hypotheses that no covariates are associated with treatment readmission. Hypothesis tests for individual covariate effects were interpreted only if this global test was statistically significant.

## Results

### Descriptive statistics

**Fig 1:**
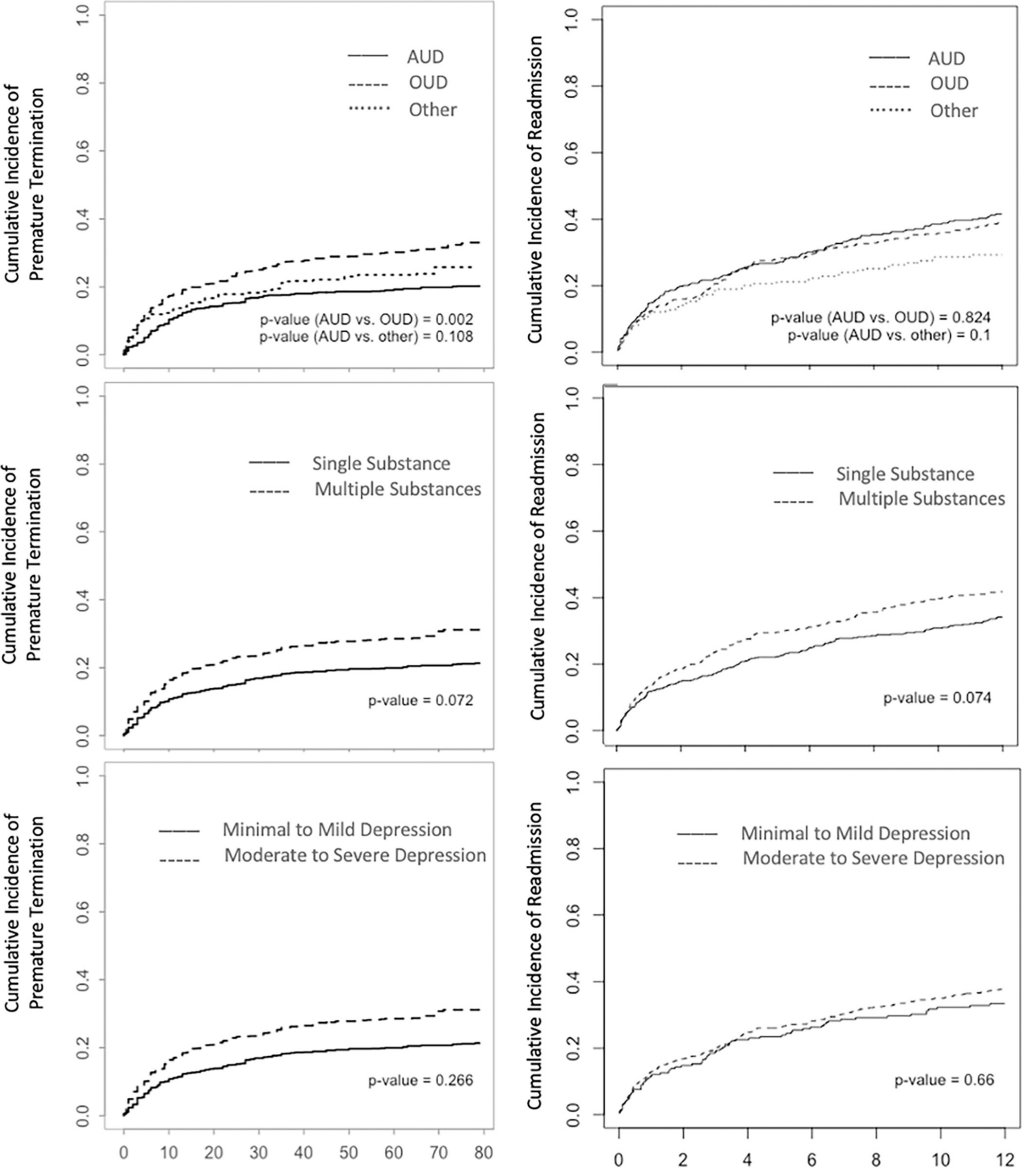
Cumulative incidence of premature treatment termination and readmission by group.

The sample had a mean PHQ-9 of 14.13 (SD = 6.19) at admission, indicative of moderate depressive symptoms, and most commonly presented with AUD (42.25%) or OUD (38.56) (see Table 1 for full sample characteristics). Being admitted with a primary OUD was associated with increased cumulative probability of premature termination over time compared to being admitted with primary AUD (*p* = 0.002) (see Fig 1).

**Table 1:** Descriptive statistics for first treatment admission.

	**Mean**	**SD**	**% Missing**
Total admissions	1.8	1.36	0
Treatment length*	31.68	48.67	0
Gap to readmission*	447	391.4	0
PHQ-9	14.13	6.19	9
Age at intake	36.5	13.92	0.9
	**N**	**%**	**% Missing**
Sex			0.3
Male	277	51.3	
Female	263	48.7	
Race			5.7
White	451	88.26	
Black	39	7.63	
Asian	2	0.39	
Bi-Racial	9	1.76	
Hispanic	4	0.78	
Other	5	0.98	
Substance use disorder			0
Alcohol	229	42.25	
Cannabis	14	2.58	
Hallucinogen	1	0.18	
Opioid	209	38.56	
Sedative	64	11.81	
Stimulant	25	4.61	
Discharge reason			
Successful completion	383	70.66	
Premature termination	146	26.94	
In treatment	13	2.4	
Treatment programs**			0
Addiction Medicine	81	4.24	
Detox	866	45.29	
Intensive outpatient	298	15.59	
Partial hospitalization	325	17.00	
Recovery Support	33	1.73	
Rehabilitation	75	3.92	
Residential	91	4.76	
Supportive living	143	7.48	

*In number of days

**treatment programs received across all admissions; a single admission frequently included a sequence of treatment programs

### Competing risks analysis for premature treatment termination

The global Wald test for the null hypothesis that no covariates are associated with premature treatment termination was statistically significant (p-value<0.001). The interaction between time-dependent depressive symptoms level and primary SUD did not significantly predict the hazard of premature treatment termination, and thus was removed from the final analysis to aid interpretation of the results and for model parsimony (Table 2). Time-dependent depressive symptoms level did not significantly predict instantaneous rate of premature treatment termination (*HR* = 1.00, *p* = 0.814). Primary SUD diagnosis at admission significantly predicted the rate of premature treatment termination; primary OUD predicted a higher hazard of premature termination compared to primary AUD (*HR* = 1.91, *p*<0.001). No other covariates significantly predicted premature treatment termination.

**Table 2:** Analysis for the premature treatment termination and treatment readmission process.

	***HR [95% CI]***	***z***	***p***
	***Premature Treatment Termination***		
PHQ-9*	0.99 [0.88, 1.10]	-0.24	0.814
SUD at treatment intake			
OUD (vs AUD)	**1.91 [1.35, 2.69]**	**3.69**	**<0.001**
Other SUD (vs AUD)	1.00 [0.60, 1.67]	0.01	0.996
Lifetime multiuse	1.16 [0.85, 1.6]	0.93	0.352
Age	0.99 [0.98, 1.01]	-0.92	0.360
Female	0.99 [0.73, 1.33]	-0.09	0.930
Caucasian	1.07 [0.65, 1.75]	0.26	0.797
	***Treatment Readmission***		
Lifetime multiuse	**1.55 [1.18, 2.06]**	**3.10**	**0.002**
SUD at treatment intake			
OUD (vs AUD)	1.15 [0.84, 1.57]	0.85	0.398
Other SUD (vs AUD)	0.69 [0.43, 1.11]	-1.55	0.122
Age	**1.01 [1, 1.02]**	**2.34**	**0.019**
Female	1.15 [0.87, 1.52]	1.01	0.311
Caucasian	0.77 [0.52, 1.13]	-1.34	0.180
Premature (vs successful) termination**	1.33 [0.99, 1.79]	1.87	0.061
PHQ-9 x Lifetime multiuse			**0.024**
PHQ-9* in Multiusers	0.98 [0.95, 1.01]	-1.38	0.169
PHQ-9* in Single substance users	1.14 [0.99, 1.32]	1.83	0.067

*Note*: Statistically significant results appear in bold

*for 5-unit increase in PHQ-9

**In the previous admission; SUD = substance use disorder, AUD = alcohol used disorder, OUD = opioid use disorder, HR = Hazard Ratio, CI = confidence interval

### Survival analysis for treatment readmission

The global Wald test for the null hypothesis that no covariates are associated with treatment readmission was statistically significant (p-value<0.001). The interaction between depressive symptoms level and lifetime substance misuse (multiple vs. single) significantly predicted treatment readmission (*p* = 0.024; Table 2); for those with single substance lifetime misuse, depressive symptoms level fell just short of significantly predicting the hazard of treatment readmission (*HR* = 1.03, *p* = 0.067), whereas the effect of depressive symptoms level did not significantly predict treatment readmission among those with lifetime misuse of multiple substances (*HR* = 0.98, *p* = 0.169). Age was positively associated with rate of readmission (*HR* = 1.01, *p* = 0.019).

## Discussion

The goal of this longitudinal study was to examine time-dependent depressive symptoms level at each admission, primary SUD at each admission, lifetime misuse of multiple substances, and their interactions as risk factors of treatment course patterns over time in a community-based SUD clinic. Consistent with our hypothesis, primary OUD diagnosis at admission significantly predicted premature termination compared to primary AUD diagnosis and this effect was large. Our work replicates and extends previous research documenting this effect [24–26] using a sophisticated longitudinal analysis, which offers stringent statistical control, and a community-based clinic sample, which offers ecological validity. Taken together, this work increases confidence in the effect of OUD diagnosis on premature termination. Premature termination may be a sign of low engagement in intensive or step-down outpatient programs [30], or may be driven by the demanding nature of OUD treatments, which require a high level of patient commitment, including regular monitoring by multiple treatment professionals. Not remaining engaged in ongoing outpatient or supportive care is a risk factor for SUD relapse [35] and future work should seek to better understand and prevent what is driving this increased premature termination risk among those with OUD.

Contrary to our hypotheses, depressive symptoms level, history of misuse of multiple substances, and their interactions did not significantly predict premature termination. This is inconsistent with several previous studies [15, 22, 23]; however, this previous work used less diverse samples, included patients with only one SUD diagnosis, and/or only used baseline measures of depression without accounting for time-dependent depressive symptoms level over time. This result is consistent with previous studies that show no relationship between depressive symptoms or depression and treatment course across a variety of study designs (e.g., studies with retrospective designs based on electronic health records to longitudinal trials), samples (e.g., local clinics or community samples of patients diagnosed with a variety of SUDs or a specific SUD diagnosis), sample sizes (ranging between 70 and 557 patients), depression measures (e.g., categorical clinical diagnosis or symptom-based scores), and treatment settings (e.g., more specialized inpatient, detoxification, residential SUD settings, and outpatient clinics) [17–21]. However, based on our study results, it is impossible to determine whether the null effect of depressive symptoms level and its interaction with history of misuse of multiple substances on SUD treatment course reflects a lack of a relationship or is a result of type II error.

Consistent with our hypotheses and previous work [17], history of misuse of multiple substances predicted readmission to treatment, indicating that more complex substance misuse histories impart risk for more frequent and faster treatment readmission. Also, as expected, there was a significant interaction between depressive symptoms level and lifetime history of substance misuse for treatment readmission, but the direction of this interaction did not support our hypothesis. Depressive symptoms level trended towards positively predicting the rate of treatment readmission, but only among those with lifetime misuse of a single substance. This effect was small but potentially of theoretical interest that needs to be further understood. Mood disorders are strong predictors of substance use symptom reoccurrence [2], which is expected to translate to faster subsequent treatment readmission. This finding may reflect that the relationship between depressive symptoms level and subsequent readmission differs as a function of substance misuse complexity. Although depressive symptoms level showed a trend of faster engagement with treatment among those with simpler misuse histories, it appears unrelated to treatment access for people with a more complex misuse history. This might reflect depressive symptoms level as a more primary driver of treatment readmission for those with a simpler substance misuse history and/or that depressive symptoms level may play less of a role (e.g., complex misuse may be a stronger contributor of readmission which may outweigh the effect of depressive symptoms), or its role may be masked (e.g., those with high depressive symptoms level and complex substance misuse do not reach care fast enough after relapse), in those with more complex misuse histories. This interaction may, in part, explain inconsistencies in previous research concerning the role of depressive symptoms on treatment course [15, 17–23].

We propose understanding the mechanisms driving this interaction effect as a prime next step in this program of research, since more complex substance misuse patterns increase likelihood for unintentional or intentional overdose fatalities, the prevalence of which are greater in the context of depressive symptoms and suicidality [2]. This finding points to an important disparity in SUD treatment utilization in a high-risk and vulnerable group, especially since depressive symptoms level, which is often a warning sign for relapse risk [10] may fail to signal risk among this higher risk group. There may be a need for concerted efforts among health care professionals and other supports to closely monitor patients with more complicated substance misuse histories for changes in mood and signs of substance use reoccurrence. This is critical given that untreated depressive symptoms can result in further problems, including increasing the potential for suicide [10] before reaching clinical care. Interestingly, age predicted treatment readmission, which has been inconsistently found in previous work [17]; thus, more research is needed to understand this effect.

This study had several limitations. Most of the patients were Caucasian with sufficient socioeconomic resources to either pay for or have insurance for treatment, which may limit generalizability. Due to low racial diversity, we were not able study racial differences. Patients received a variety of different treatment modalities, which may increase generalizability of findings, but could potentially confound results. We only had data from one treatment facility and could not ascertain whether patients were admitted to other clinics during the follow-up period. Rates of lifetime history of misuse of multiple substances were slightly lower (at 38%) than may be expected by previous estimates (e.g., 55–64% [36, 37]), which may reflect an underreporting self-report bias. Although the PHQ-9 is a well-supported measure of depressive symptoms [31, 32], other measures may contribute complementary information to better understand effects on treatment course. Depressive symptoms level was measured at the beginning of treatment; including measurements at the end of treatment would have helped us understand depressive symptom changes secondary to treatment and would provide a more proximal measure to characterize if unresolved depressive symptoms contributed to premature termination and/or readmission.

Overall, this work extends upon previous findings of this nature, by providing evidence utilizing a rigorous time-dependent, recurrent event and competing risks survival analysis approach and a real-world clinic-based sample, controlling for covariates that may have confounded this past literature. Thus, the impact of this study is that it provides a more stringent test and support for the finding that OUD diagnosis places one at risk for premature treatment termination, which is particularly important, as those with OUD who terminate prematurely are at higher risk for substance use relapse, which is associated with higher risk of overdose and death [2]. Since primary diagnosis is readily available in clinics, this is a low-cost/high-reward strategy without additional staff burden. Lifetime misuse of multiples substances, age, and premature termination in the previous treatment admission may be important to predict return to treatment; these variables can be used to focus close monitoring on patients who may most benefit from such efforts. This work also provides one explanation for the mixed results concerning how depressive symptoms level influences SUD treatment course and patterns by identifying substance misuse history complexity as a moderator of this relationship. Depressive symptoms level, which is easily assessed and tracked over time, may be an important early warning sign for those who have a history of only misusing one substance that returning to treatment may be warranted or necessary, but may be a less useful warning sign for those with more complex misuse histories. The current study utilized a real-world clinic-based sample, providing complementary findings to better controlled, but less ecologically valid, clinical trial approaches. We believe that assessing and testing these complex relationships in real-world clinic samples has potential to directly influence real-world clinics and treatment settings. With advancements in statistical modeling, we propose that this study can serve as an example of how future research should continue to utilize more complex models to attempt to replicate previously documented effects, with the long-term goal of better characterizing risks, designing and planning treatment, and supporting SUD patients and their families.
